# A pilot study of Ipilimumab in patients with stage IV melanoma receiving palliative radiation therapy

**DOI:** 10.1186/2051-1426-2-S3-P75

**Published:** 2014-11-06

**Authors:** Susan Knox, Sunil Reddy, Susan M Hiniker, Susan Swetter, Lei Shura, Holden T Maecker

**Affiliations:** 1 Stanford University Radiation Oncology, Stanford, CA, USA; 2 Stanford University Medical Oncology, Stanford, CA, USA; 3 Stanford University Dermatology, Stanford, CA, USA; 4 Stanford University, Stanford, CA, USA

## Hypothesis

Local palliative radiation therapy can be safely used in combination with anti-CTLA-4 immunotherapy. This therapy may have the potential to enhance the induction of systemic anti-melanoma immune responses, which will inhibit growth and kill melanoma cells in sites of established metastasis outside of the radiation therapy field more effectively than with anti-CTLA-4 alone.

## Objectives

The primary objective is to assess the safely of combining Ipilimumab with palliative radiation therapy in patients with unresectable Stage IV melanoma. Secondary objectives include assessment of 1) induction of anti-melanoma immune responses using laboratory correlative studies of immune responses to melanoma antigens, 2) comparison of tumor response rates and duration of response at unirradiated tumor sites in patients with Stage IV disease with historically stage IV patients treated with Ipilimumab alone.

## Methods

This is a single institution, open-label, pilot study of 20-25 patients with unresectable (*or disseminated*) stage IV melanoma requiring palliative radiation therapy. Patients are treated with Ipilimumab (3mg/kg) every 3 weeks for a total of 4 treatments. Palliative radiation therapy to 1-2 sites of disease is initiated within 5 days of the first dose of Ipilimumab. Tumor imaging studies are obtained at baseline, 2-4 weeks following the 4^th ^dose of Ipilimumab, and every 3 months until disease progression. Both Response Evaluation Criteria in Solid Tumors (RECIST) and response by immune response criteria are used to assess tumor responses. Patients are monitored for safety, and a variety of immune response parameters are measured before and during the treatment period. These include enumeration of major cell subsets as well as myeloid-derived suppressor cells (MDSC), and antigen-specific T cell responses by intracellular cytokine staining.

## Results

This is an ongoing study. Seventeen patients with metastatic melanoma have been treated to date. The combined treatment has been well tolerated with no unexpected toxicities, and no apparent exacerbation of either radiation or Ipilimumab associated toxicities. Tumor and immune response data will be presented.

## Conclusions

The combination of palliative radiation therapy and Ipilimumab has been well tolerated in patients with metastatic melanoma. Tumor and immune response data are being collected and analyzed, and conclusions of this ongoing trial will be presented.

